# Supplementary material for: Juvenile Heat Tolerance in Wheat for Attaining Higher Grain Yield by Shifting to Early Sowing in October in South Asia
Source: Genes (Basel). 2021 Nov 18;12(11):1808. doi: 10.3390/genes12111808 (PMC8622066; doi:10.3390/genes12111808)
Supplement: Supplementary file 1 [file genes-12-01808-s001.zip › genes-1425096-supplementary.pdf]

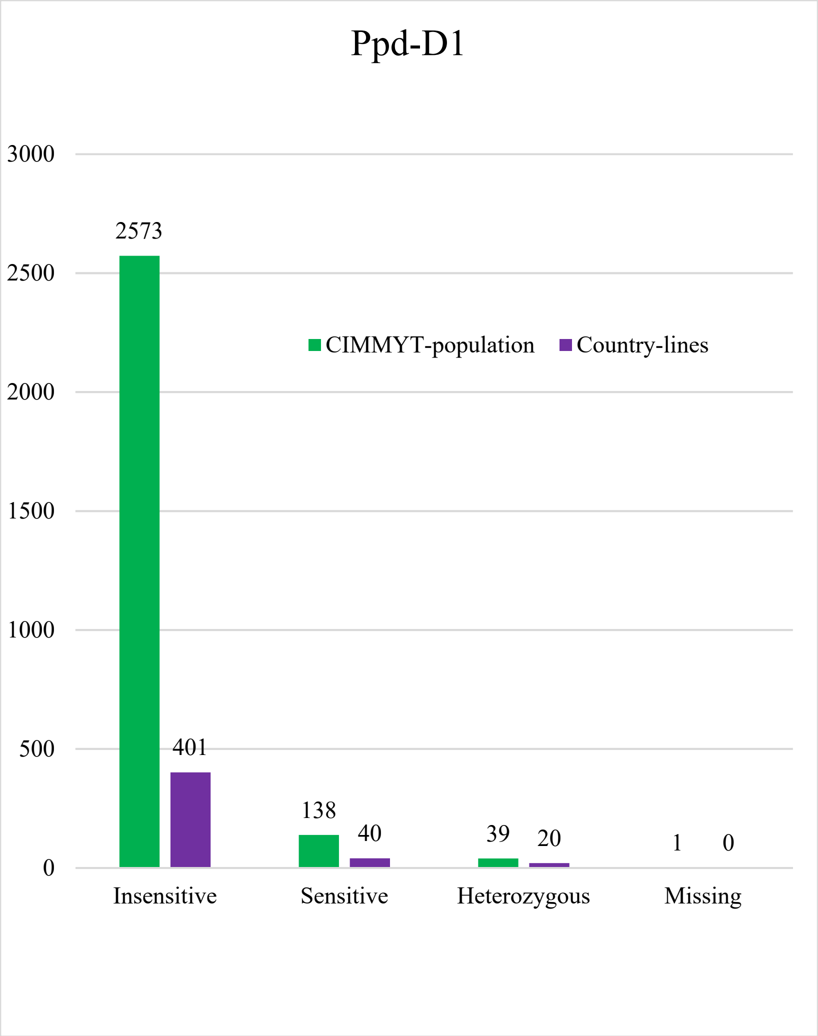

Figure S1. Genotyping results for *Ppd-D1*

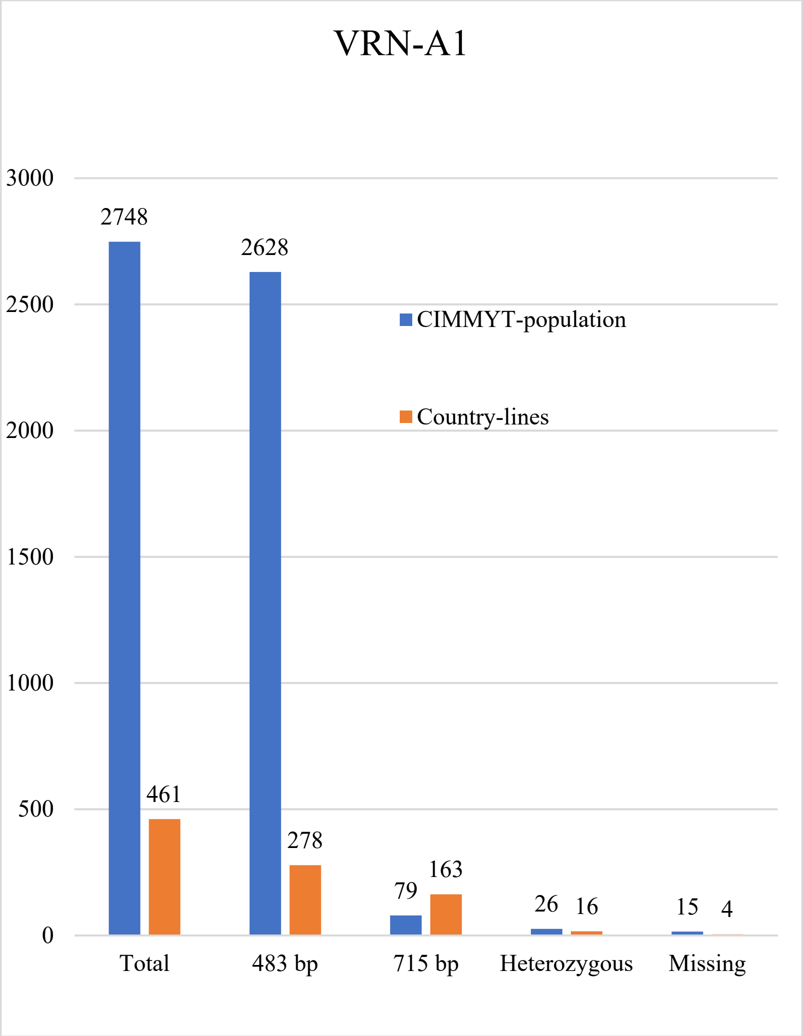

Figure S2. Genotyping results for *Vrn-A1*

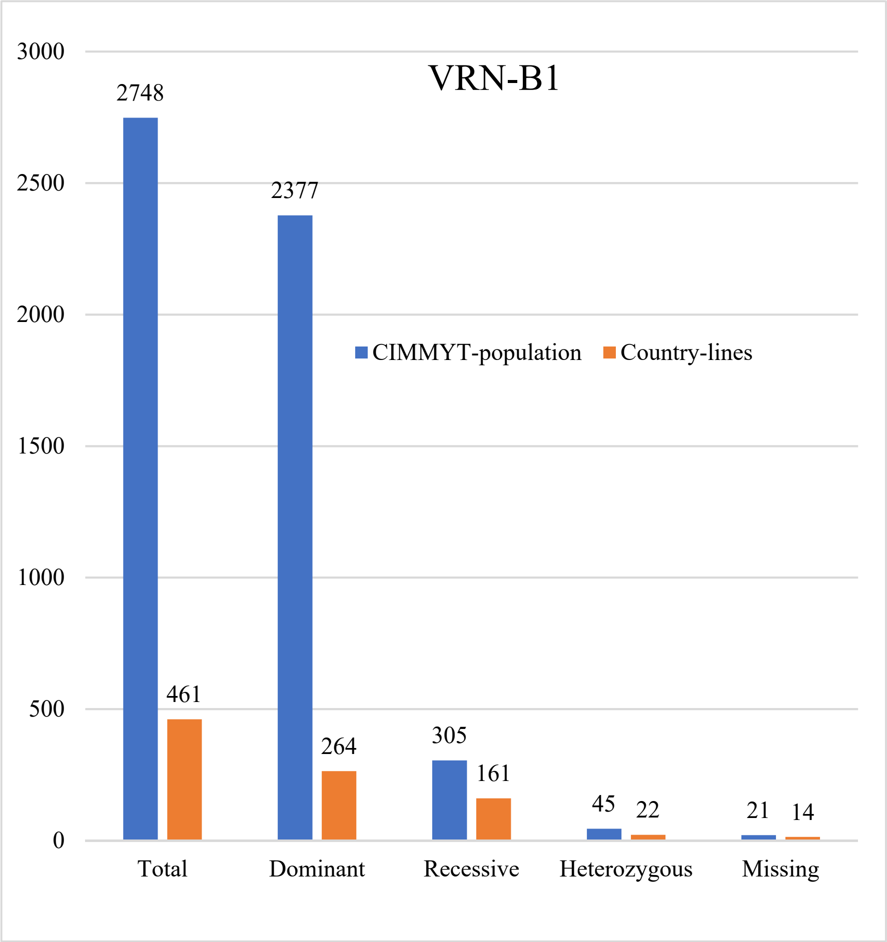

Figure S3. Genotyping results for Vrn-B1

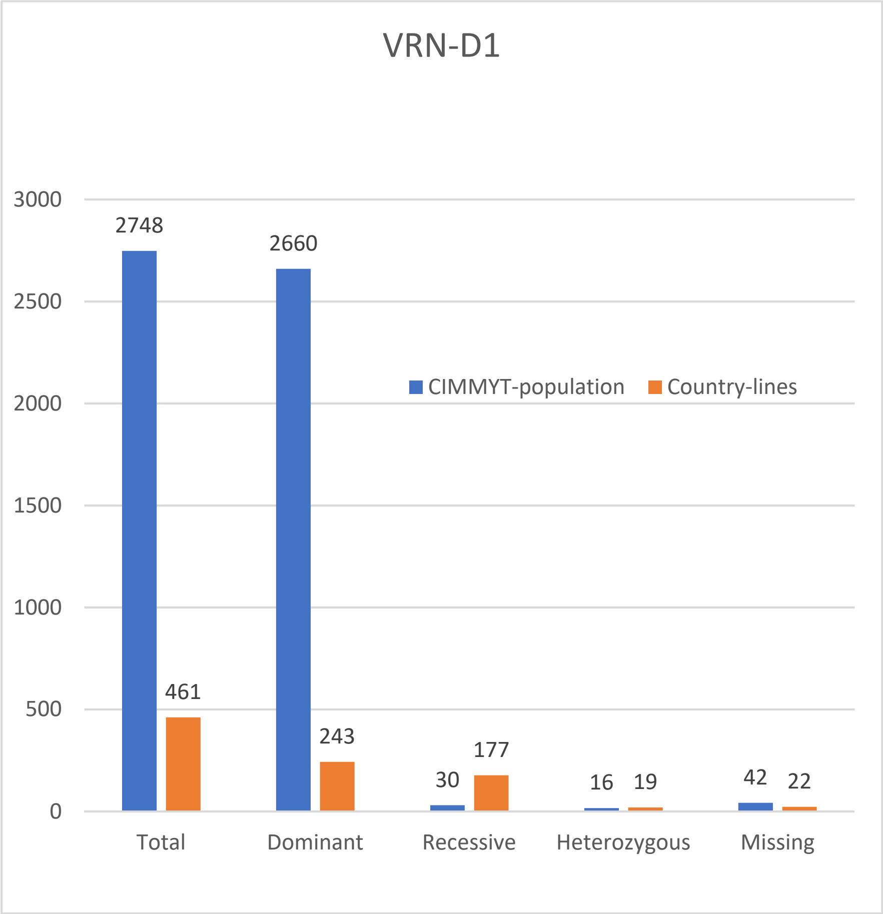

Figure S4. Genotyping results for Vrn-D1

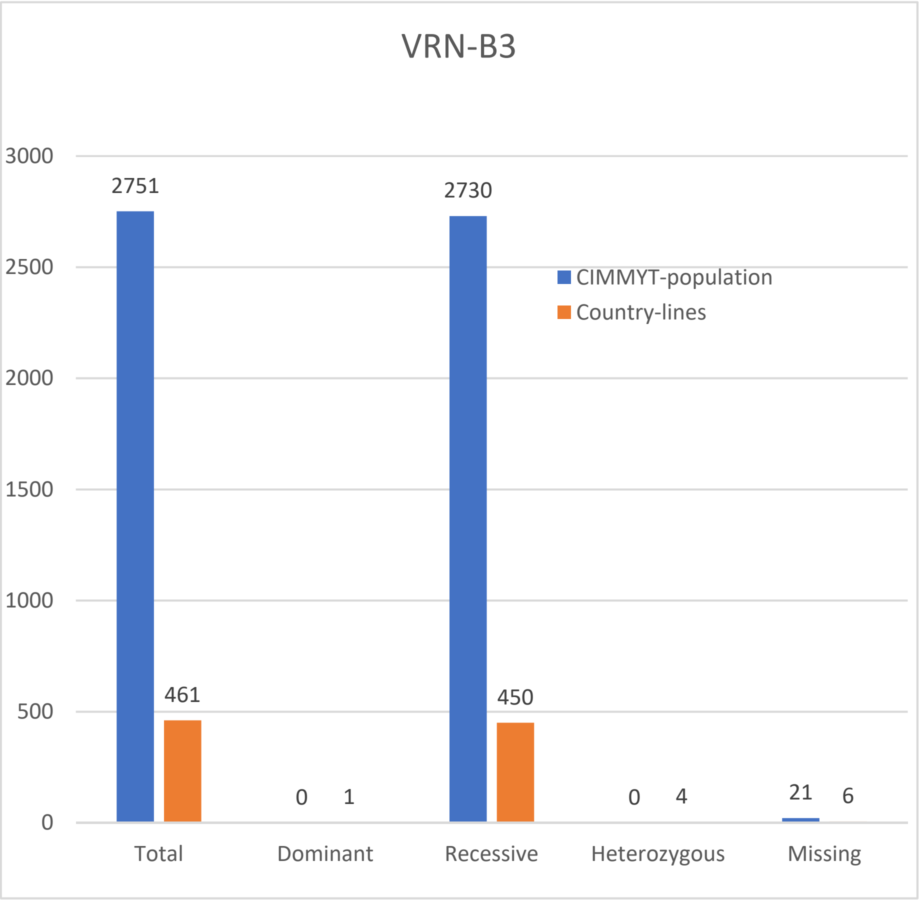

Figure S5. Genotyping results for Vrn-B3 in CIMMYT and country lines

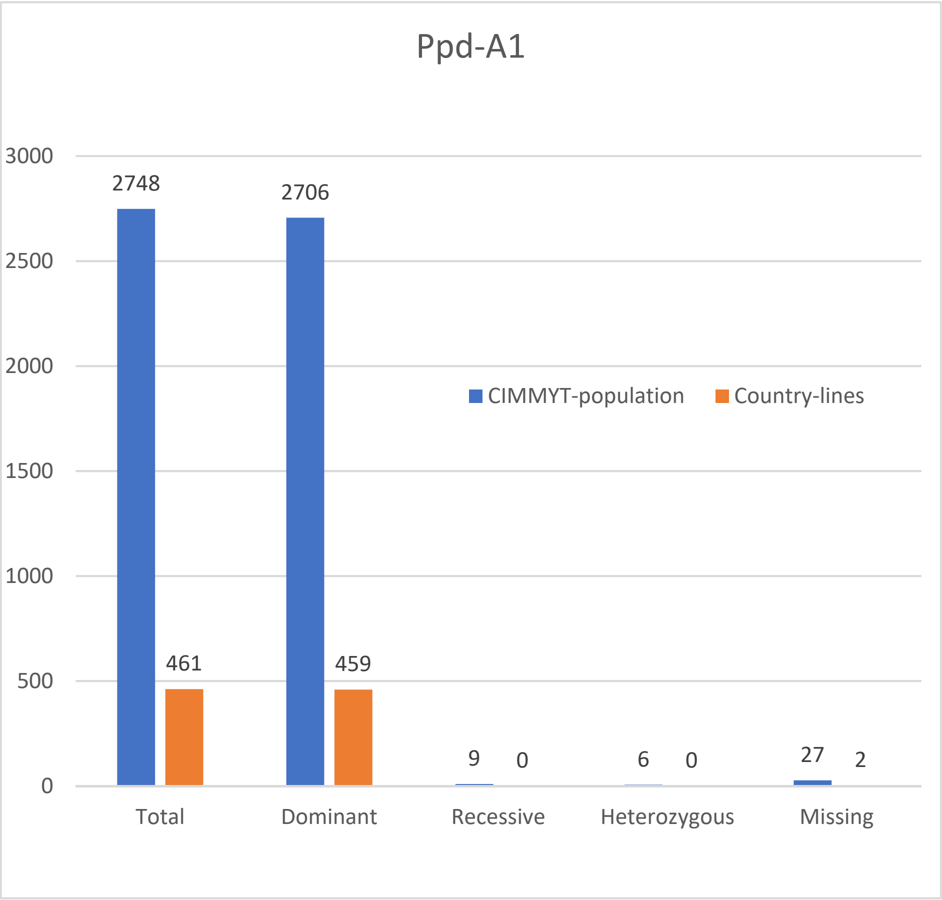

Figure S6. Genotyping results for Ppd-A1

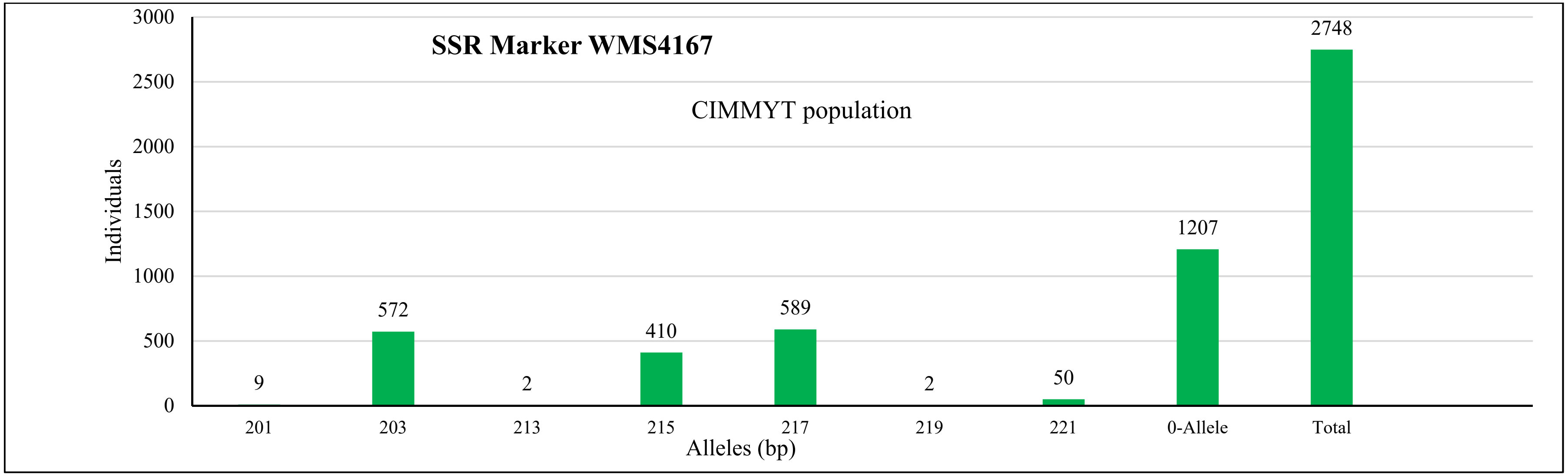

Figure S7. Genotyping of SSR-marker WMS4167 (linked to *Ppd-B1*) in the CIMMYT lines

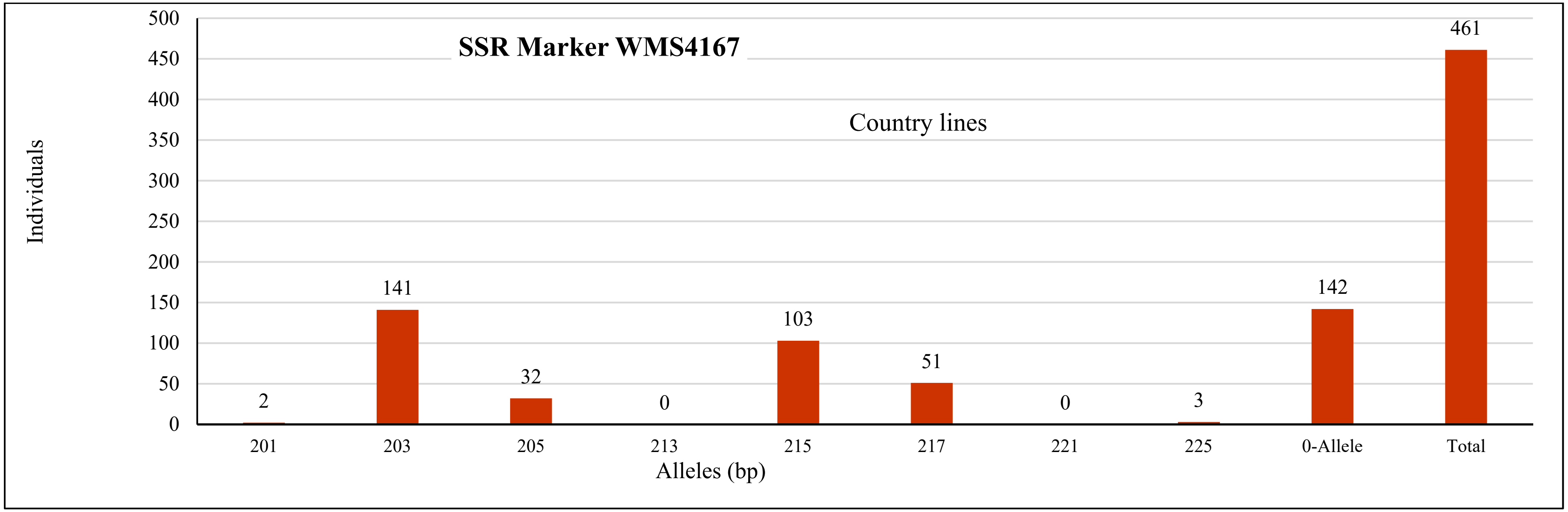

Figure S8. Genotyping of SSR-marker WMS4167 (linked to *Ppd-B1*) in the country lines

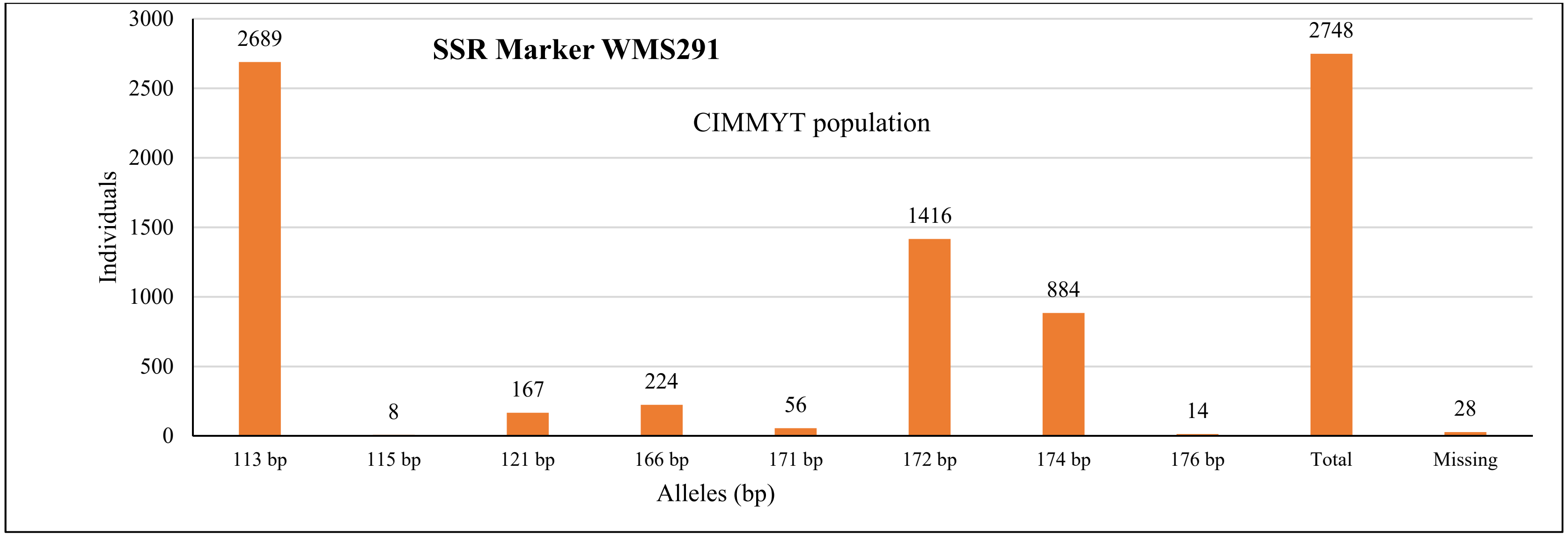

Figure S9. Genotyping of SSR-marker WMS291 (linked to*Vrn-A2*) in the CIMMYT lines

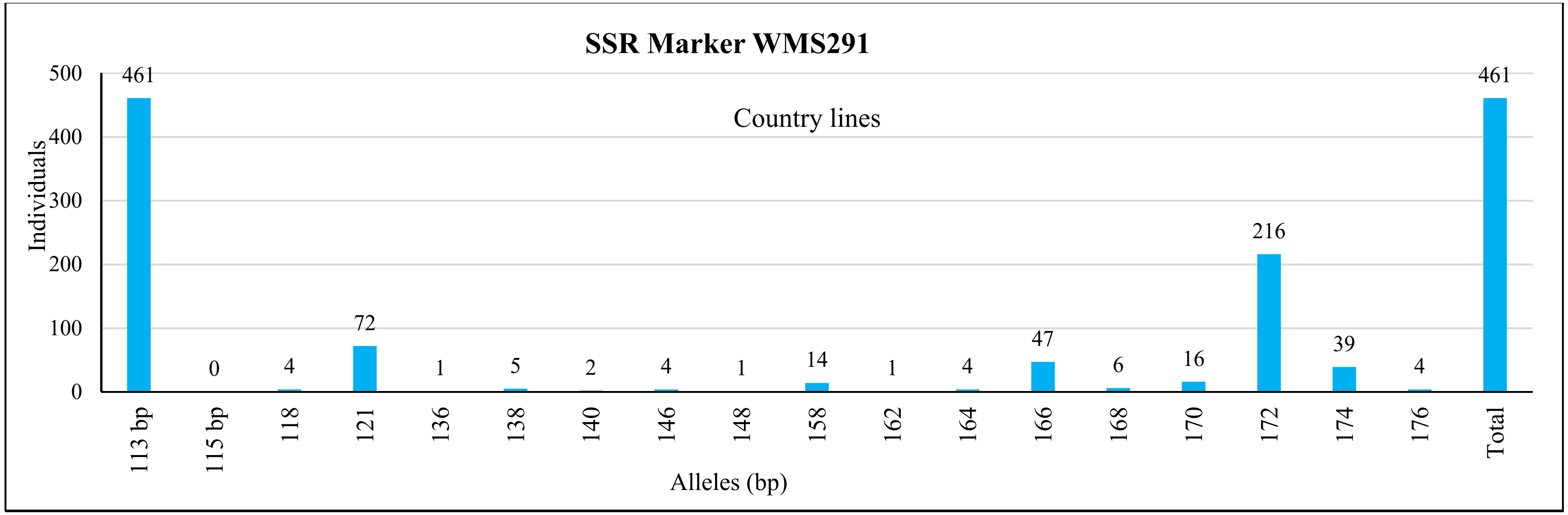

Figure S10. Genotyping of SSR-marker WMS291 (linked to *Vrn-A2*) in the country lines

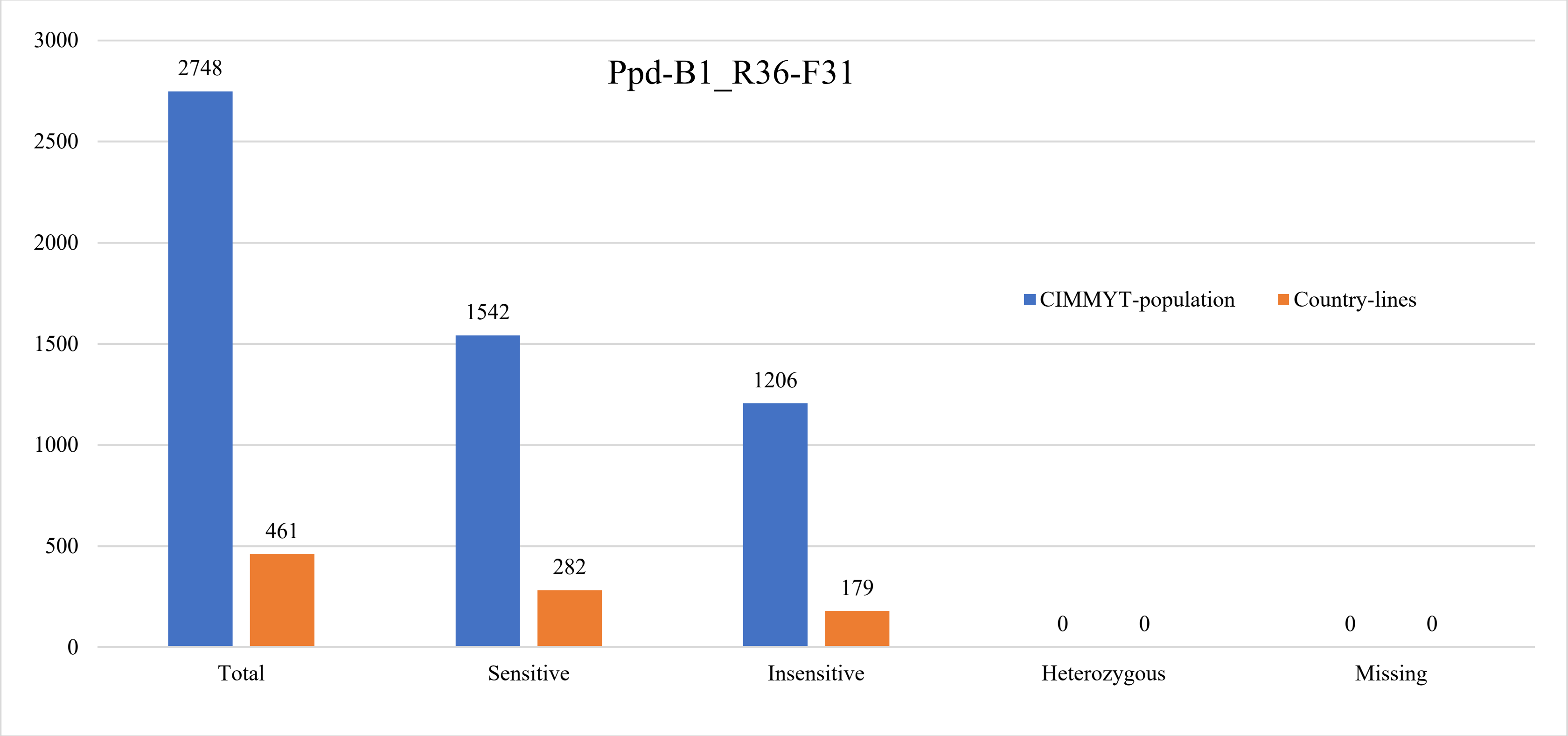

Figure S11. Genotyping results for Ppd-B1_R36-F31

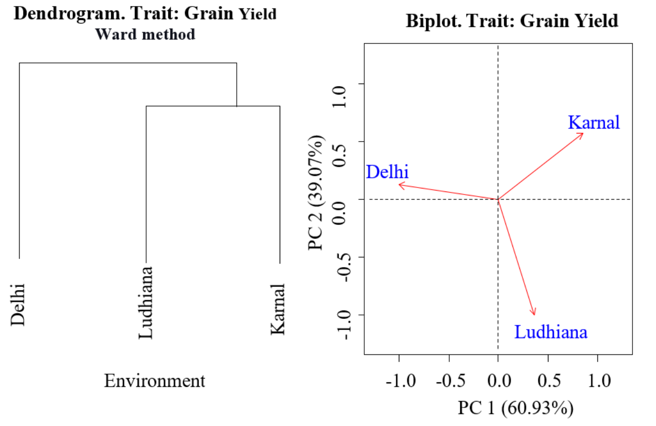

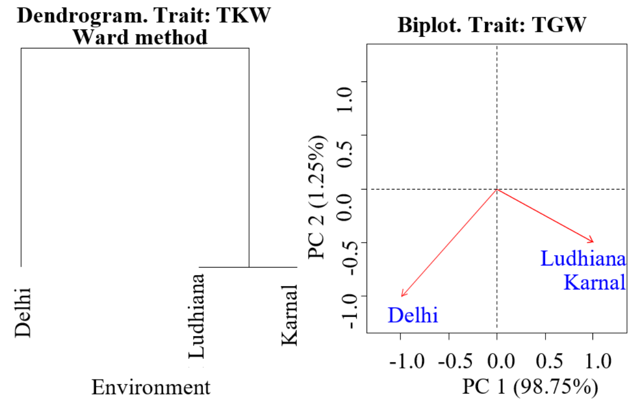

Grain yield	Thousand Grain Weight

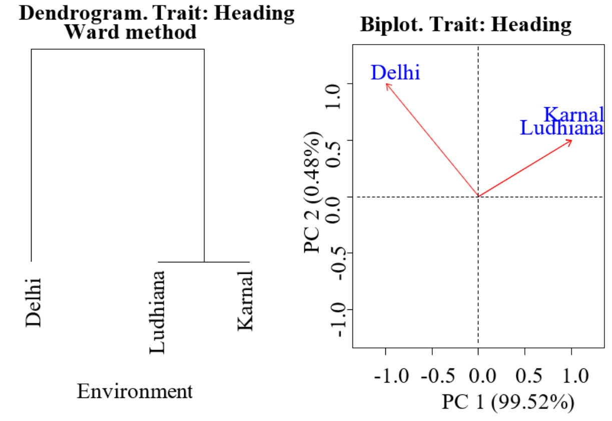

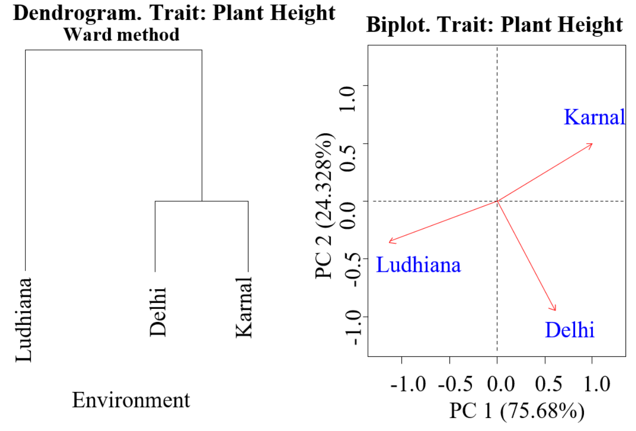

Days to heading	Plant height

Figure S12. Biplot and dendrogram for three locations of NWPZ of India for grain yield and yield traits when diverse 50 wheat lines were tested under October sowing of 2013-14

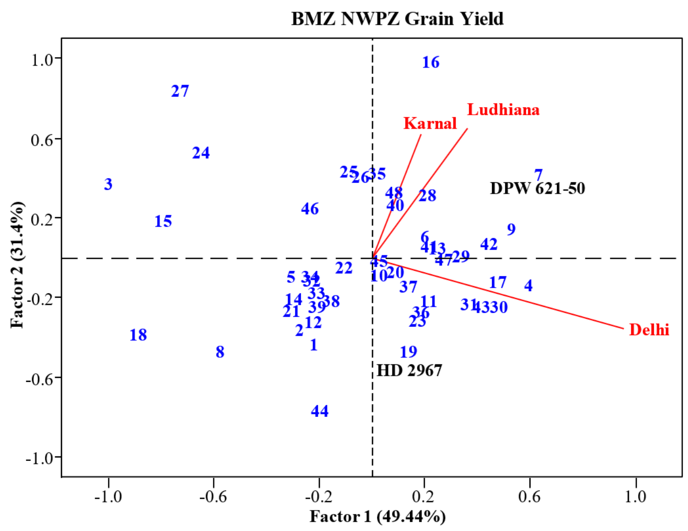

Grain yield

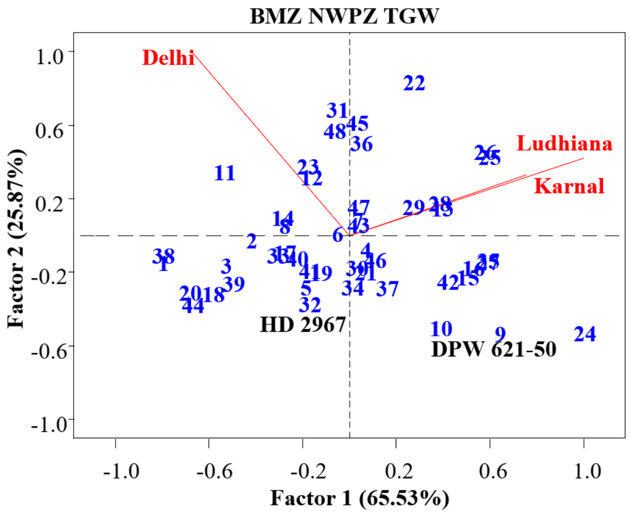

Thousand Grain Weight

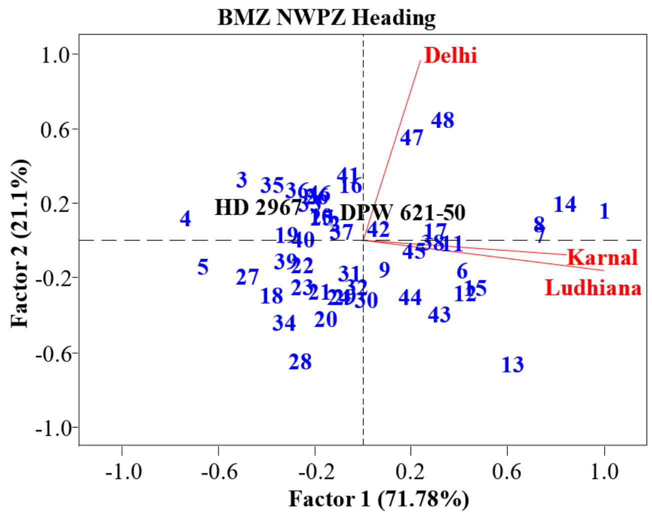

Days to heading

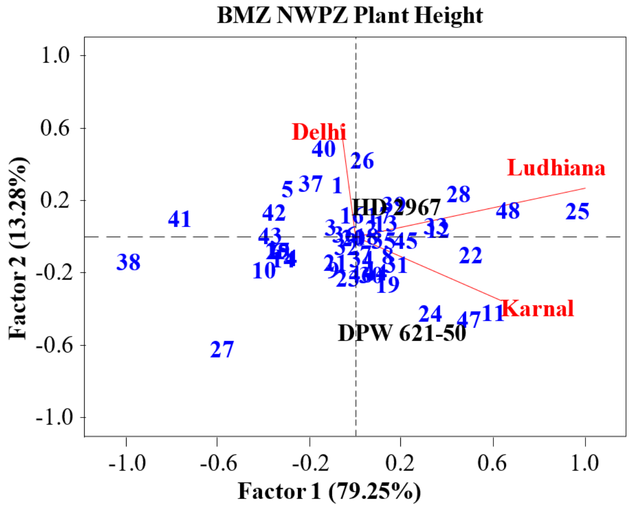

Plant height

Figure S13. Biplot for grain yield and yield traits for 50 lines tested under October sowing of 2013-14 in three locations of NWPZ of India

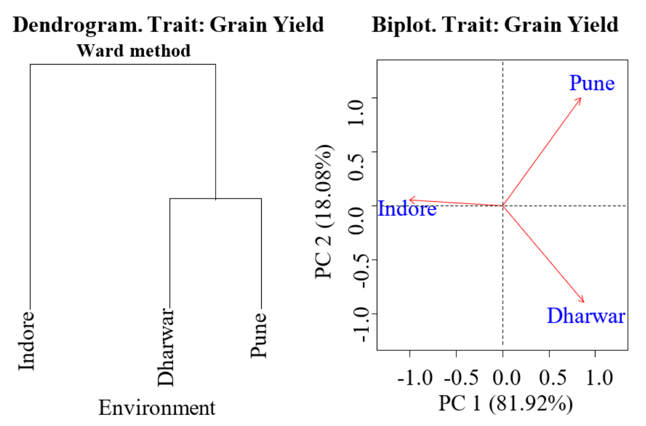

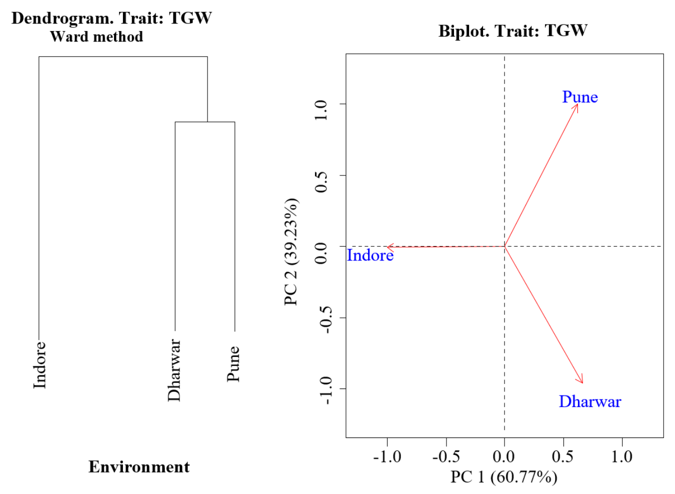

Grain yield

Thousand Grain Weight

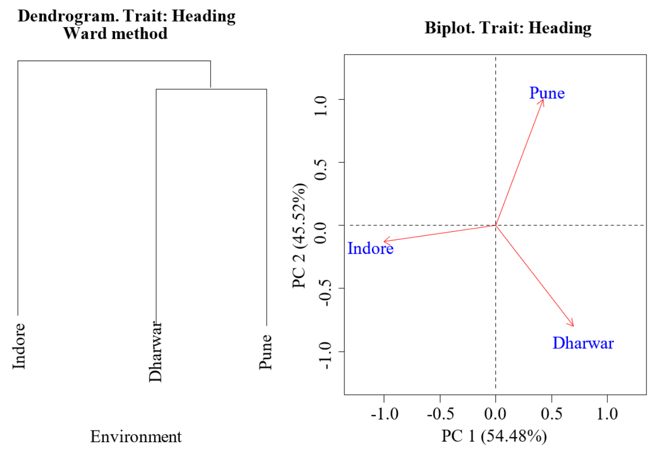

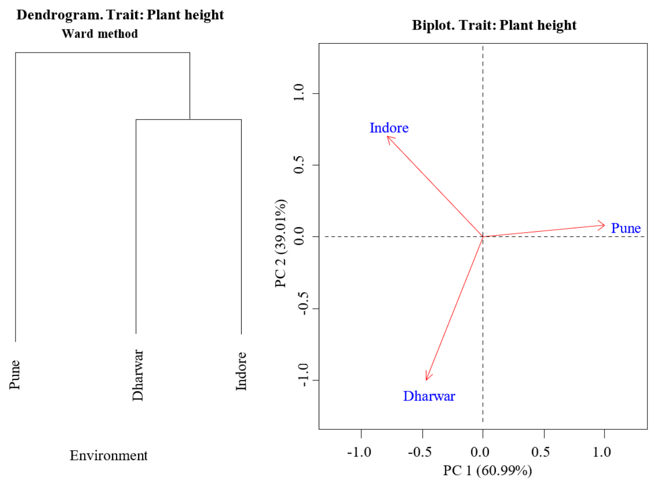

Days to heading

Plant height

Figure S14. Biplot and dendrogram for three locations of CPZ of India for grain yield and yield traits when diverse 50 wheat lines were tested under October sowing of 2013-14

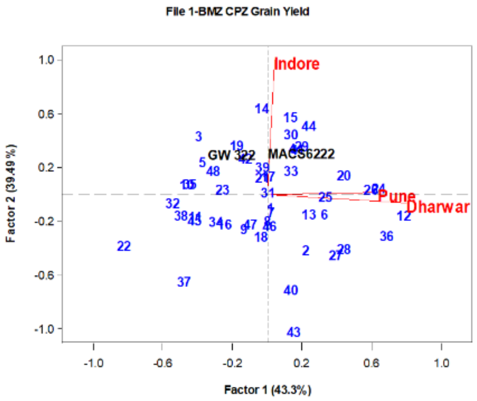

Grain yield

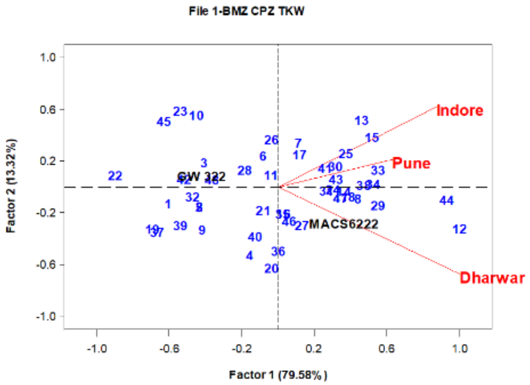

Thousand Grain Weight

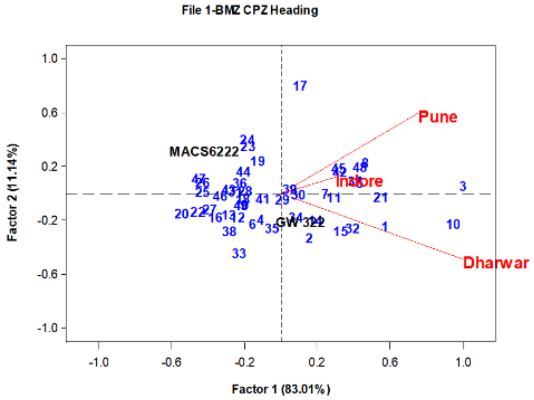

Days to heading

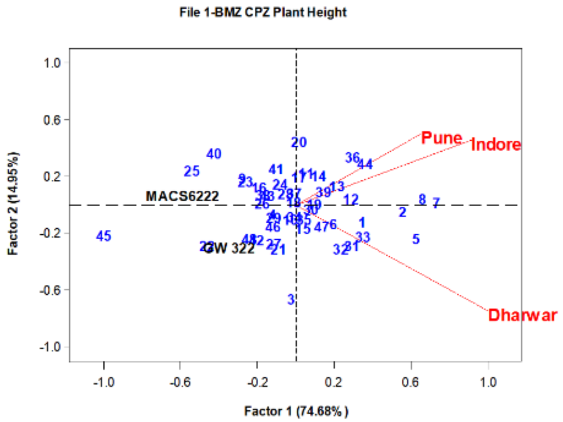

Plant height

Figure S15. Biplot for grain yield and yield traits for 50 lines tested under October sowing in three locations of CPZ of India in 2013-14

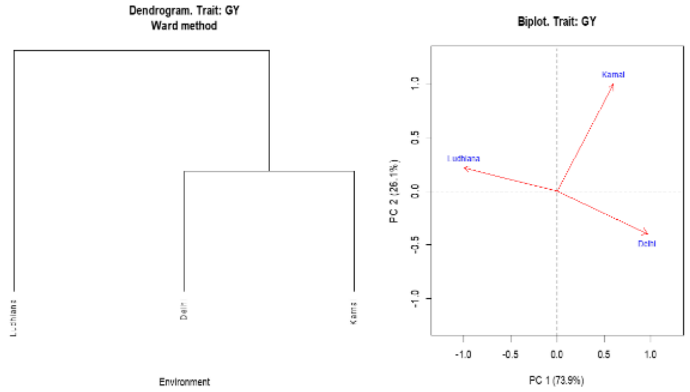

Grain yield

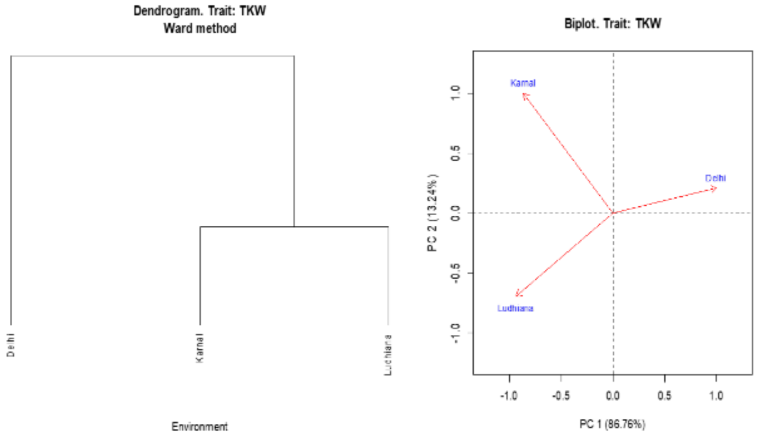

Thousand Grain Weight

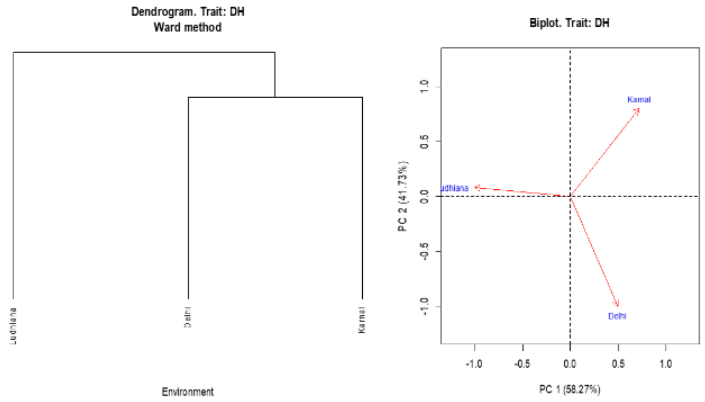

Days to heading

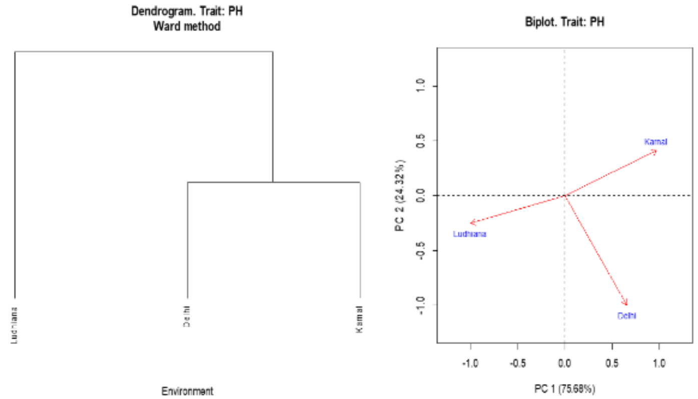

Plant height

Figure S16. Biplot and dendrogram for three locations of NWPZ of India for grain yield and yield traits when diverse 30 wheat lines were tested under October sowing of 2014-15

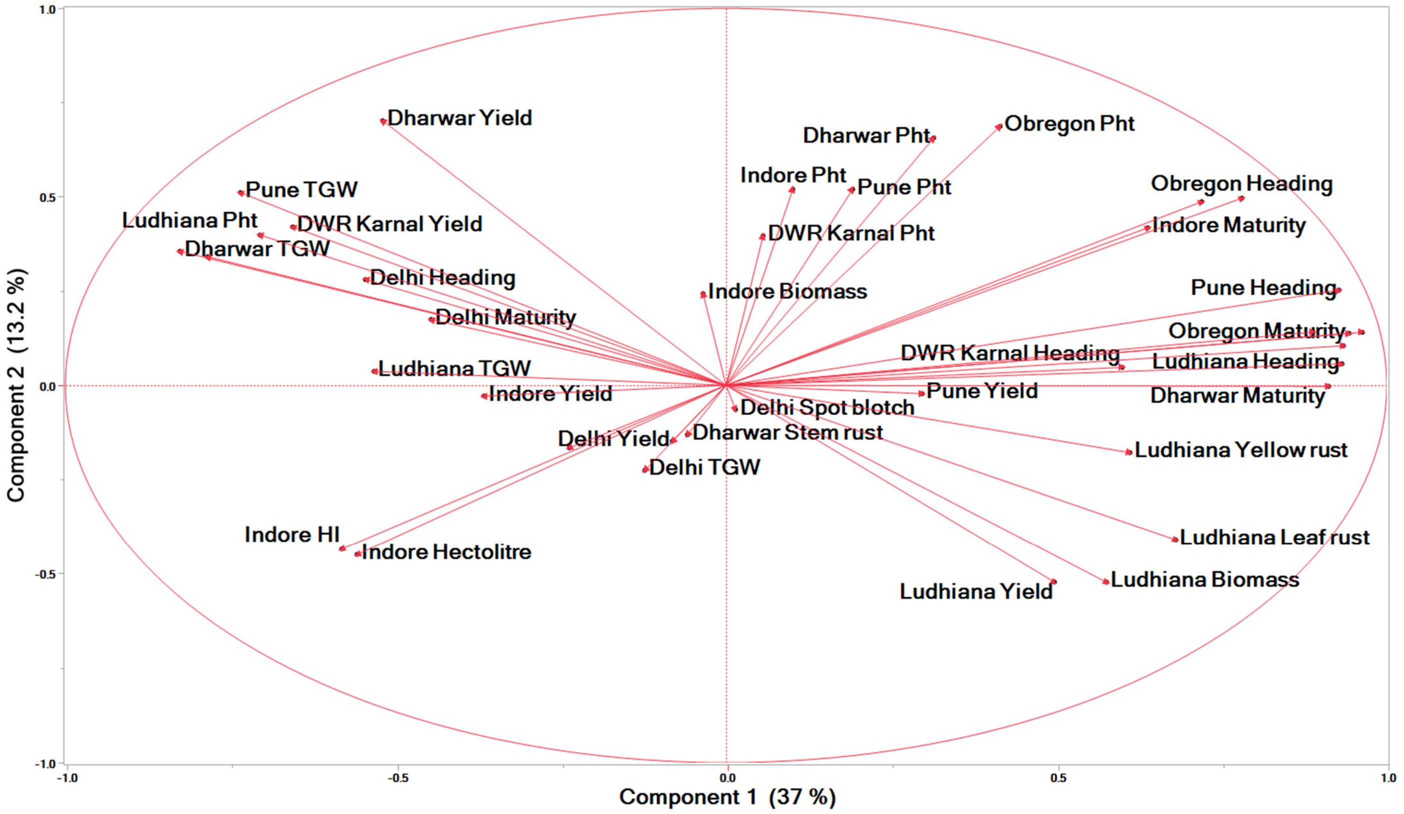

Figure S17: PCA plot of the traits in the different environments

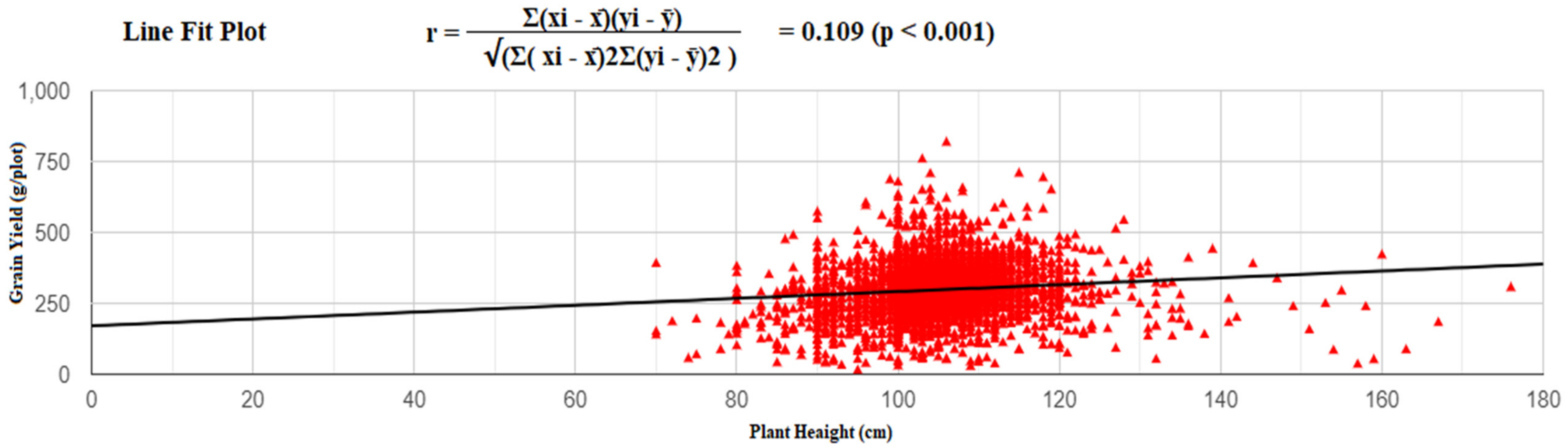

Figure S18 Line Fit Plot for plant height and grain yield for 3222 lines evaluated under early sown condition

Table S1: Details of lines with pedigree, origin source, selection history, unique GID and other information.

	**CIMMYT-population**		**Country-lines**	
**Ppd-D1**				
Total	2748	100%	461	100.00%
Insensitive	2573	93.60%	401	86.99%
Sensitive	138	5.02%	40	8.68%
Heterozygous	39	1.42%	20	4.34%
Missing	1	0.04%	0	0.00%
**VRN-A1**				
Total	2748	100%	461	100%
Insensitive	2628	95.63%	278	60.30%
Sensitive	79	2.87%	163	35.36%
Heterozygous	26	0.95%	16	3.47%
Missing	15	0.55%	4	0.87%
**VRN-B**1				
Total	2748	100%	461	100.00%
Insensitive	2377	86.50%	264	57.27%
Sensitive	305	11.10%	161	34.92%
Heterozygous	45	1.64%	22	4.77%
Missing	21	0.76%	14	3.04%
**VRN-D1**				
Total	2748	100%	461	100.00%
Insensitive	2660	96.80%	243	52.71%
Sensitive	30	1.09%	177	38.39%
Heterozygous	16	0.58%	19	4.12%
Missing	42	1.53%	22	4.77%
**VRN-B3**				
Total	2748	100%	461	100.00%
Insensitive	2751	100.11%	1	0.22%
Sensitive	0	0.00%	450	97.61%
Heterozygous	2730	99.34%	4	0.87%
Missing	0	0.00%	6	1.30%
**Ppd-A1**				
Total	2748	100%	461	100.00%
Insensitive	2706	98.47%	459	99.57%
Sensitive	9	0.33%	0	0.00%
Heterozygous	6	0.22%	0	0.00%
Missing	27	0.98%	2	0.43%

Table S2: Type, number and frequency of alleles based on DNA analysis of markers associated with Vrn nd Ppd genes

	**CIMMYT-population**		**Country-lines**	
**Ppd-D1**				
Total	2748	100%	461	100.00%
Insensitive	2573	93.60%	401	86.99%
Sensitive	138	5.02%	40	8.68%
Heterozygous	39	1.42%	20	4.34%
Missing	1	0.04%	0	0.00%
**VRN-A1**				
Total	2748	100%	461	100%
Insensitive	2628	95.63%	278	60.30%
Sensitive	79	2.87%	163	35.36%

Heterozygous	26	0.95%	16	3.47%
Missing	15	0.55%	4	0.87%
**VRN-B**1				
Total	2748	100%	461	100.00%
Insensitive	2377	86.50%	264	57.27%
Sensitive	305	11.10%	161	34.92%
Heterozygous	45	1.64%	22	4.77%
Missing	21	0.76%	14	3.04%
**VRN-D1**				
Total	2748	100%	461	100.00%
Insensitive	2660	96.80%	243	52.71%
Sensitive	30	1.09%	177	38.39%
Heterozygous	16	0.58%	19	4.12%
Missing	42	1.53%	22	4.77%
**VRN-B3**				
Total	2748	100%	461	100.00%
Insensitive	2751	100.11%	1	0.22%
Sensitive	0	0.00%	450	97.61%
Heterozygous	2730	99.34%	4	0.87%
Missing	0	0.00%	6	1.30%
**Ppd-A1**				
Total	2748	100%	461	100.00%
Insensitive	2706	98.47%	459	99.57%
Sensitive	9	0.33%	0	0.00%
Heterozygous	6	0.22%	0	0.00%
Missing	27	0.98%	2	0.43%

Table S3: Associations using a two-sided t-test and the p-values for the t-test used to indicate significance of the association among heading and alleles of Ppd and Vrn genes

Trait	Term	Estimate	Std Error	t Ratio	Prob>|t| (p-value)	Significance of the association
Delhi Heading	Intercept	90.92	2.95	30.8	4.79E-176	***
Delhi Heading	Ppd-D1a sensitive	-0.95	0.56	-1.71	8.82E-02	NS
Delhi Heading	VRN A1 715 bp	-0.83	1.27	-0.65	5.13E-01	NS
Delhi Heading	VRN D1 dominant	-4.61	2.23	-2.07	3.86E-02	NS
Delhi Heading	VRN-B1 dominant	2.20	1.08	2.03	4.21E-02	NS
Dharwar Heading	Intercept	62.80	2.13	29.52	7.04E-164	***
Dharwar Heading	Ppd-D1a sensitive	5.67	0.40	14.05	3.63E-43	***
Dharwar Heading	VRN A1 715 bp	-8.66	0.92	-9.44	8.53E-21	***
Dharwar Heading	VRN D1 dominant	-1.39	1.61	-0.87	3.86E-01	NS
Dharwar Heading	VRN-B1 dominant	-2.49	0.78	-3.19	1.42E-03	**
DWR Karnal Heading	Intercept	115.53	3.08	37.45	1.34E-242	***
DWR Karnal Heading	Ppd-D1a sensitive	5.14	0.58	8.82	2.20E-18	***
DWR Karnal Heading	VRN A1 715 bp	-7.99	1.33	-6.01	2.19E-09	**
DWR Karnal Heading	VRN D1 dominant	-1.83	2.33	-0.78	4.33E-01	NS
DWR Karnal Heading	VRN-B1 dominant	-3.30	1.13	-2.92	3.52E-03	NS
Indore Heading	Intercept	129.20	1.74	74.41	0.00E+00	***
Indore Heading	Ppd-D1a sensitive	2.95	0.33	9.02	3.72E-19	***
Indore Heading	VRN A1 715 bp	0.47	0.76	0.62	5.35E-01	NS
Indore Heading	VRN D1 dominant	-3.44	1.31	-2.63	8.58E-03	NS
Indore Heading	VRN-B1 dominant	-0.69	0.63	-1.09	2.77E-01	NS
Ludhiana Heading	Intercept	107.87	2.55	42.28	1.31E-292	***
Ludhiana Heading	Ppd-D1a sensitive	4.73	0.48	9.8	2.89E-22	***
Ludhiana Heading	VRN A1 715 bp	-5.14	1.09	-4.71	2.68E-06	**
Ludhiana Heading	VRN D1 dominant	-1.65	1.91	-0.86	3.87E-01	NS
Ludhiana Heading	VRN-B1 dominant	-0.22	0.94	-0.23	8.17E-01	NS
Obregon Heading	Intercept	87.48	3.25	26.93	6.13E-140	***
Obregon Heading	Ppd-D1a sensitive	7.81	0.62	12.69	9.31E-36	***
Obregon Heading	VRN A1 715 bp	-11.94	1.40	-8.52	2.66E-17	***
Obregon Heading	VRN D1 dominant	-2.58	2.45	-1.05	2.92E-01	NS
Obregon Heading	VRN-B1 dominant	-2.88	1.19	-2.42	1.56E-02	NS
Pune Heading	Intercept	63.07	2.40	26.23	1.03E-133	***
Pune Heading	Ppd-D1a sensitive	6.34	0.45	13.95	1.31E-42	***
Pune Heading	VRN A1 715 bp	-9.58	1.04	-9.24	5.36E-20	***
Pune Heading	VRN D1 dominant	-3.03	1.81	-1.67	9.52E-02	NS
Pune Heading	VRN-B1 dominant	-0.56	0.88	-0.63	5.28E-01	NS
